# Objective Structured Assessment of Technical Skills in Mastoidectomy Using 3D‐Printed Temporal Bones

**DOI:** 10.1002/lary.70583

**Published:** 2026-05-08

**Authors:** Vanessa Helou, Lucien Khalil, Philip L. Perez, Andrew A. McCall, Noel Jabbour

**Affiliations:** ^1^ Department of Otolaryngology‐Head and Neck Surgery University of Pittsburgh Pittsburgh Pennsylvania USA; ^2^ Department of Head and Neck Surgery University of California Los Angeles Los Angeles California USA

**Keywords:** 3D‐printed temporal bone, competency‐based medical education, mastoidectomy, OSATS, otolaryngology training

## Abstract

**Objective:**

To evaluate the use of three‐dimensional (3D) printed temporal bone models combined with final product analysis as an objective structured assessment of technical skills tool to assess mastoidectomy performance across levels of otolaryngology residency training.

**Methods:**

In this prospective observational study conducted at a single academic otolaryngology residency program, 32 residents performed 64 mastoidectomies on 3D‐printed temporal bone models over a three‐year period. Three expert faculty independently and anonymously evaluated each performance using a validated 14‐item final product analysis checklist. Statistical analyses included linear regression, ANOVA, Mann–Whitney U, Wilcoxon signed‐rank tests, and intraclass correlation coefficient (ICC) for inter‐rater reliability.

**Results:**

Median scores improved steadily with training level, from 35.0 in postgraduate year (PGY) 1 to 53.7 in PGY 5 out of 70. Linear regression demonstrated a significant positive association between both training level and duration in residency with performance. Senior residents (PGY 4–5, median score 54.0) significantly outperformed junior residents (PGY 1–3, median score 38.0; *p* = 0.012). Across all training levels, median scores improved from 43.2 on the first attempt to 52.5 on the third, though this difference was not statistically significant. Inter‐rater reliability was excellent (ICC = 0.9210, *p* < 0.0001). A proficiency cutoff score of 45/70 was established using the contrasting groups method.

**Conclusion:**

The consistency of 3D‐printed temporal bones judged by final product analysis allows for fair, objective, and reliable assessment for formative and summative evaluations. By longitudinally tracking skill improvement and differentiating performance across training levels, it supports the transition to competency‐based surgical education.

**Level of Evidence:**

N/A.

## Graphical abstract

This study validated a 3D‐printed temporal bone model combined with final product analysis as an objective assessment tool for mastoidectomy surgical skills in otolaryngology residency. Performance scores improved significantly with training level, with excellent inter‐rater reliability. This approach supports competency‐based surgical education through standardized, reliable skills assessment.

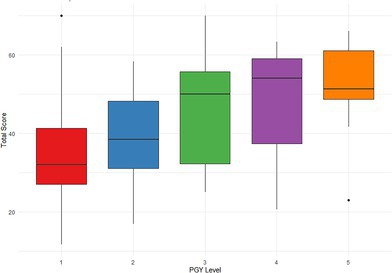

## 1 Introduction

Mastoidectomy training is a cornerstone of otolaryngology residency as it develops essential surgical skills for managing chronic ear disease and related pathologies. Mastery of mastoidectomy requires proficiency in complex three‐dimensional anatomy, fine motor control, and spatial judgment—skills necessary to avoid injury to critical structures such as the facial nerve and inner ear.

Historically, surgical education rested on the education model of “see one, do one, teach one,” yet this paradigm lacked objective benchmarks and reproducibility. In response, graduate medical education has shifted toward competency‐based medical education (CBME), where learners must demonstrate clearly defined skills and milestones before advancing. CBME places emphasis on frequent, structured assessments and individualized feedback, ensuring that each trainee attains proficiency rather than merely accumulating case numbers [1, 2]. As such, Objective Structured Assessment of Technical Skills (OSATS) tools have been explored in mastoidectomy training. These tools include global rating scales, task‐specific checklists, and final product analysis to provide reliable and valid measures of resident performance throughout training [3, 4]. Their use enables formative feedback, targeted remediation, and the establishment of benchmarks for unsupervised practice, which is essential for effective surgical education and patient safety [3, 5, 6].

Traditional mastoidectomy training with cadaveric temporal bones, while invaluable for anatomic realism, presents several challenges, including anatomical variability, limited accessibility, and high costs. To address these limitations, several methods for training and assessing skills were developed in the operating room, virtual reality, or three‐dimensional (3D) printed temporal bone models [7]. 3D‐printed temporal bone models represent a promising solution, offering the ability to produce anatomically accurate and consistent models that allow for controlled, repeatable training and transfer of skills [8, 9]. A systematic review by Frithioff et al. highlighted that while 3D‐printed models are widely reported and well‐received, the overall educational quality of supporting studies remains low, with most evidence limited to trainee and expert opinion [8]. This study proposes an objective assessment tool using 3D‐printed temporal bone models to assess mastoidectomy performance throughout residency training.

## 2 Materials and Methods

### 2.1 Study Design

Between November 2022 and July 2024, otolaryngology residents from all training years (postgraduate year (PGY) 1 through PGY‐5) performed mastoidectomy with facial recess approach using 3D‐printed temporal bone models as part of their benchmarking skills assessment. Each resident completed one to three attempts over the course of the study. Temporal bone models were constructed to replicate normal anatomy based on high‐resolution CT data, as previously validated by our group [10]. Key features include patient‐specific anatomical accuracy, realistic drillability, and low production cost. All residents consented to participate and did not receive financial compensation. This study was deemed to be exempt from review by the University of Pittsburgh Institution Review Board (study #19090297).

### 2.2 Final Product Analysis Checklist

Each 3D‐printed temporal bone was assessed, anonymously and independently, by three expert faculty members who regularly perform mastoidectomy. A validated final‐product analysis checklist by Zirkle et al. was used [11]. The checklist consists of 14 items, divided into two main domains: 5 items are related to the drilling defects in critical structures and 9 items related to the identification of the cortical mastoid anatomical limits and quality of mastoidectomy. Each item is scored on a 5‐point Likert scale, with scoring criteria tailored to the nature of the task. The maximum score per mastoidectomy is 70 points. The tool scores the drilling defects in five anatomic areas: posterior canal wall, tegmen, sigmoid sinus, semicircular canals, and ossicles, and the limits of the cortical mastoid identified. For drilling defects, a score of 5 indicates no drilling defect, and lower scores correspond to progressively larger defects, with a score of 1 indicating a drilling defect larger than 4 mm. A score of “not applicable” indicates that the structures were not reached and thus the drilling defect score could not be assessed. These items were scored as 0, as failure to reach the relevant structure was considered indicative of inadequate surgical progression. The second domain of the checklist is based on the identification of the cortical mastoid anatomical limits (posterior canal wall, tegmen, sigmoid sinus, sino‐dural angle, and antrum entered), quality of mastoidectomy (gradual sloping edges, straightness of edges, appropriate depth of cavity), and the overall quality of the mastoidectomy. For the second domain, a score of 5 indicates that the task was completed to full satisfaction, and lower scores correspond to decreasing task completion and satisfaction. The sum score was calculated for each resident as the sum of their scores on the 14 items from each expert scoring. The average score of the three total scores from each expert scoring was calculated.

### 2.3 Statistical Analyses

Descriptive statistics were used to summarize performance scores, levels of training, and days in residency at the time of assessment. Residents were divided into two groups based on their level of training: junior residents (PGY1–PGY3) and senior residents (PGY4–PGY5). Median scores and interquartile range (IQR) were calculated for each PGY level and training group.

A linear regression analysis was conducted to assess the relationship between level of training and total performance score. Differences in total scores across PGY levels were evaluated using one‐way analysis of variance (ANOVA). The Mann–Whitney U test was used to compare total scores and individual checklist items between junior and senior residents, given the nonparametric distribution of checklist data. Wilcoxon signed‐rank tests were used to compare performance scores across multiple attempts by the same resident.

Pearson correlation analysis was performed to evaluate the relationship between individual checklist items and the total score. Correlation strength was interpreted using the following thresholds: values between 0.00 and 0.30 were considered weak, 0.31–0.50 moderate, 0.51–0.70 strong, 0.71–0.90 very strong, and values greater than 0.90 were interpreted as extremely strong correlations.

Inter‐rater and intra‐rater reliability among the three expert graders were assessed using the intraclass correlation coefficient (ICC), using a two‐way random‐effects model with absolute agreement. The contrasting groups method was used to determine a proficiency cutoff score by identifying the intersection point between the distributions of performance scores for novice and experienced residents [12]. Statistical significance was set at *p* < 0.05.

## 3 Results

A total of 64 3D‐printed temporal bones, drilled by 32 residents, were each assessed by three expert graders. Inter‐rater reliability among the graders was excellent (ICC = 0.921, *p* < 0.0001). Intra‐rater reliability was excellent (ICC = 0.910, *p* < 0.001). Of the 64 temporal bones assessed, 13 (20%) were drilled by PGY 1, 14 (22%) by PGY 2, 14 (22%) by PGY 3, 13 (20%) were drilled by PGY 4, and 10 (16%) by PGY 5.

**FIGURE 1:**
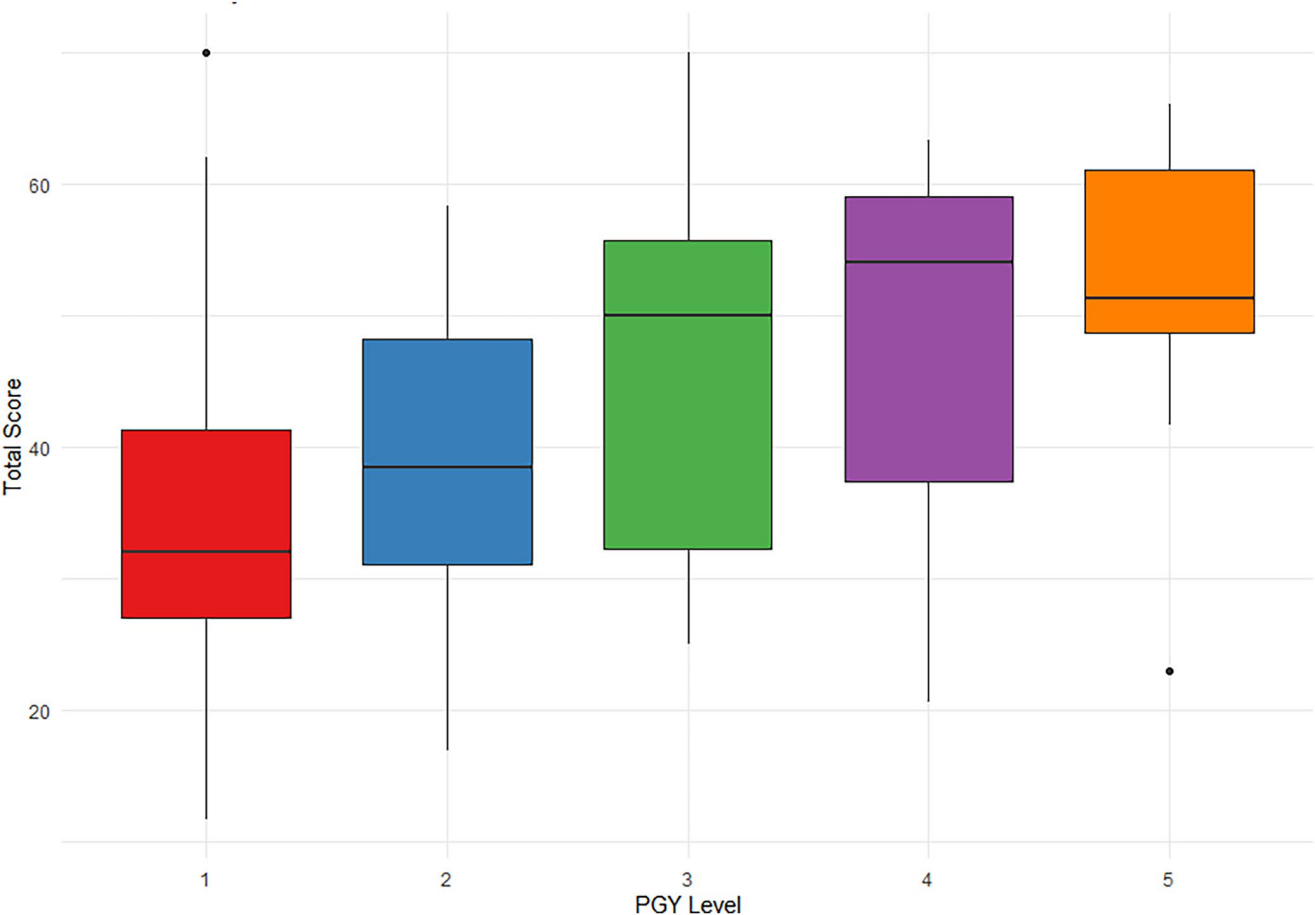
Median mastoidectomy performance scores by postgraduate year (PGY). Boxes represent the interquartile range, with the horizontal line indicating the median; whiskers denote the range excluding outliers, which are shown as individual points. Overall differences across PGY levels were statistically significant (one‐way ANOVA, *F* = 2.66, *p* = 0.041). [Color figure can be viewed in the online issue, which is available at www.laryngoscope.com]

**FIGURE 2:**
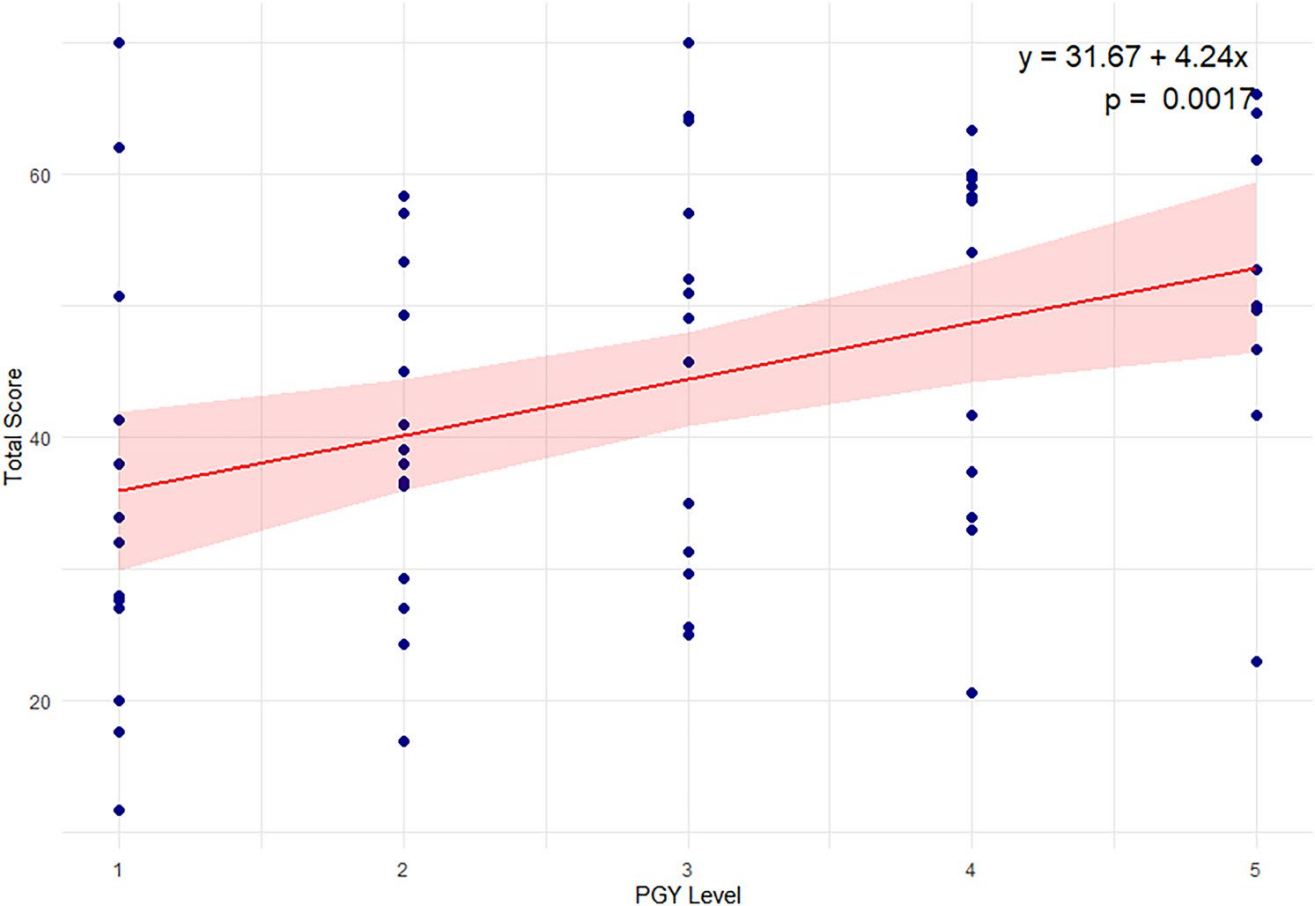
Association between training level and mastoidectomy performance. Each point represents an individual participant's total score by postgraduate year (PGY). The solid line denotes the line of best fit, and the shaded area indicates the 95% confidence interval. [Color figure can be viewed in the online issue, which is available at www.laryngoscope.com]

**FIGURE 3:**
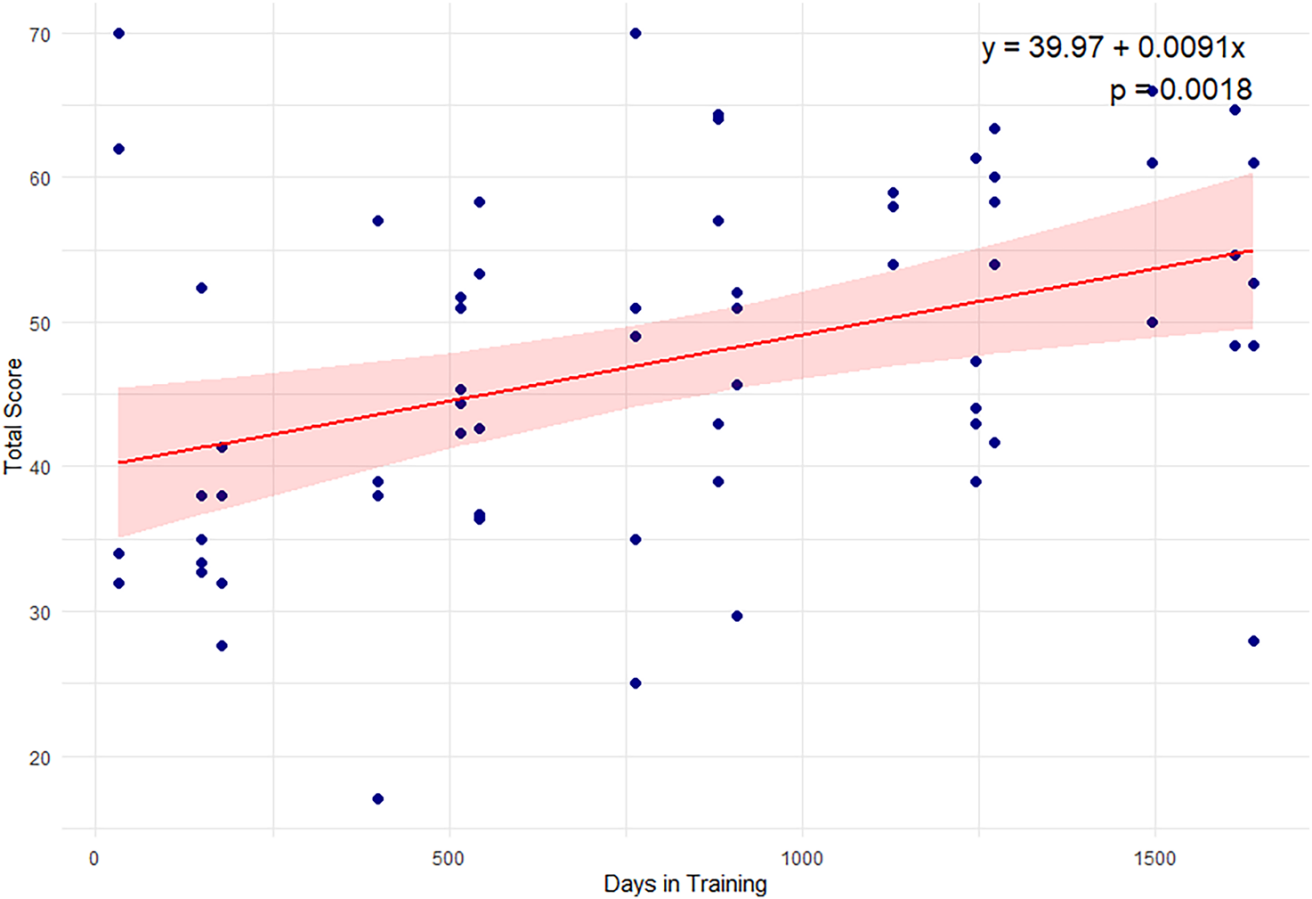
Association between days in training at assessment and mastoidectomy performance. Each dot shows an individual participant's total score by training days. The solid line represents best fit, with shaded area representing the 95% confidence interval. [Color figure can be viewed in the online issue, which is available at www.laryngoscope.com]

Median scores improved across training levels, ranging from 35.0 (IQR, 32.7–41.3) in PGY1 to 53.7 (IQR, 48.8–61.0) in PGY5, out of a maximum score of 70. There was a statistically significant overall difference in scores across PGY levels (ANOVA: *F*(4, 59) = 2.66, *p* = 0.041; Figure 1). Linear regression analysis revealed a significant positive association between level of training and performance (*β* = 4.24, *p* = 0.002; Figure 2). A similar positive association was found between the number of days in residency and performance score (*β* = 0.0091, *p* = 0.002; Figure 3).

When 3D‐printed temporal bones were grouped into junior (PGY1–PGY3, *n* = 23) and senior (PGY4–PGY5, *n* = 41) levels, senior residents demonstrated significantly higher performance, with a median total score of 54.0 (IQR, 41.7–59.8) compared to 38.0 (IQR, 28.0–51.0) in junior residents (*p* = 0.012). Seven checklist items showed statistically significant differences between the two groups, based on the Wilcoxon rank sum test: posterior canal wall limit (*p* = 0.023), sino‐dural angle (*p* = 0.016), antrum entered (*p* = 0.024), gradual sloping edges (*p* = 0.029), straightness of edges (*p* = 0.006), appropriate cavity depth (*p* = 0.015), and overall quality (*p* = 0.009). None of the drilling defect items showed statistically significant differences between groups. Median scores with corresponding IQRs are summarized in Table 1.

**TABLE 1:** Comparison of median mastoidectomy scores between junior and senior residents on each checklist item a standardized checklist.

Checklist item	Scores, median (IQR)		*p* ^a^
	Junior residents (*N* = 23)	Senior residents (*N* = 41)	
Drilling defects			
Posterior canal wall	3.7 (2.0–5.0)	4.3 (3.3–4.8)	*0.252*
Tegmen	5.0 (3.3–5.0)	5.0 (4.0–5.0)	*0.687*
Sigmoid	5.0 (3.0–5.0)	5.0 (4.2–5.0)	*0.282*
Semicircular Canals	3.3 (0.0–5.0)	5.0 (3.3–5.0)	*0.083*
Ossicles	0.0 (0.0–5.0)	3.3 (1.7–5.0)	*0.056*
Limits of mastoid			
Posterior canal wall	2.7 (2.0–3.7)	4.0 (2.2–4.3)	***0.023***
Tegmen	3.0 (2.0–3.7)	3.7 (3.0–4.0)	*0.113*
Sigmoid sinus	3.3 (2.0–4.0)	3.7 (2.8–4.0)	*0.104*
Sino‐dural angle	2.7 (2.0–3.0)	3.3 (2.5–4.0)	***0.016***
Antrum entered	2.0 (1.3–3.3)	3.7 (2.2–4.0)	***0.024***
Gradual sloping edges	3.0 (2.0–3.7)	3.3 (3.0–4.0)	***0.029***
Straightness of edges	2.7 (2.3–3.3)	4.0 (3.0–4.3)	***0.006***
Appropriate depth of cavity	2.0 (1.3–3.7)	3.7 (2.5–4.2)	***0.015***
Overall quality	2.3 (2.0–3.3)	3.3 (2.7–4.0)	***0.009***
Total score	38.0 (28.0–51.0)	54.0 (41.7–59.8)	***0.004***

*Note:* Bold indicates statistically significant *p*‐values. All *p*‐values are listed in italics.

Abbreviation: IQR, interquartile range.

^a^ *p*‐values were calculated based on Wilcoxon rank sum test.

All checklist items demonstrated strong to extremely strong correlations with the total score, indicating close alignment between individual components and overall performance. Notably, items such as depth of cavity (*r* = 0.88), antrum entered (*r* = 0.84), posterior canal wall limit (*r* = 0.83), and semicircular canal drill (*r* = 0.83) showed very strong correlations. The overall quality item exhibited an extremely strong correlation (*r* = 0.96). The summary of the correlation between checklist items and total scores is provided in Table S1.

**FIGURE 4:**
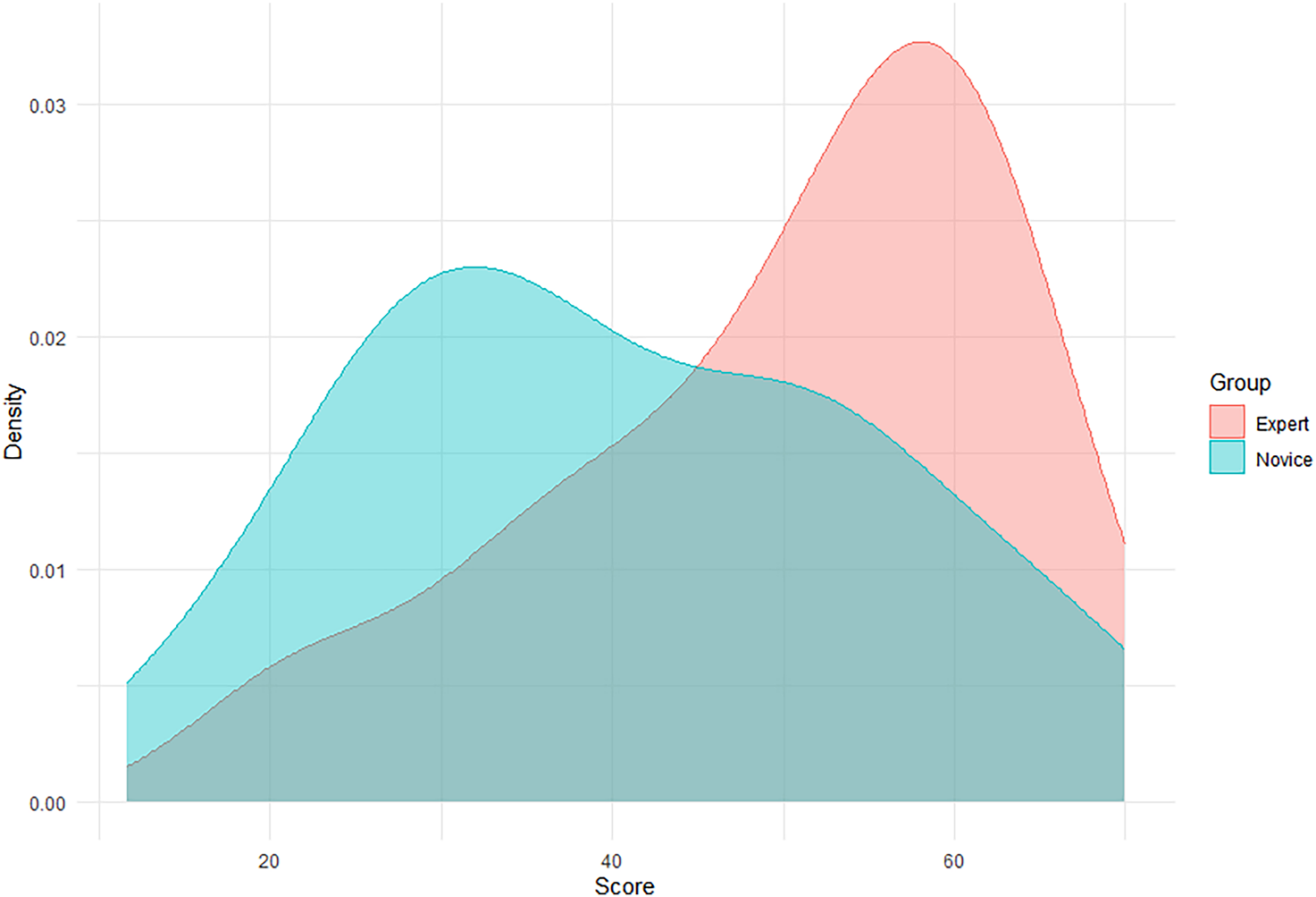
Contrasting groups method for establishing proficiency. Curves represent density plots estimating score distributions for expert and novice residents. Their intersection identifies the proficiency cutoff score, determined as 45 out of a maximum 70 points. [Color figure can be viewed in the online issue, which is available at www.laryngoscope.com]

Across all training levels, median performance scores for the same residents improved from 43.2 on the first attempt to 52.5 on the third attempt; however, this change was not statistically significant. The average time interval between attempts was 307 days (range 222–392 days). The contrasting groups method was used to set an expected proficiency score by determining the intercept between the distribution of performance scores of novices and experienced residents. A score of 45 was considered the expected proficiency score (Figure 4).

## 4 Discussion

This study reinforces the value of 3D‐printed temporal bone models combined with structured assessment tools for evaluating mastoidectomy performance in otolaryngology residency training. We demonstrated that our objective assessment tool reliably differentiated performance across training levels and showed strong inter‐rater reliability. As expected, performance improved progressively with increasing level of training, as evidenced by significantly higher median scores in senior residents compared to junior residents, and a positive linear association between PGY level and performance score. This supports the validity of the tool in measuring mastoidectomy surgical skill acquisition over time. These findings are consistent with prior studies showing that 3D‐printed temporal bone models, when paired with validated OSATS‐based tools, can differentiate levels of surgical experience, support formative and summative evaluation, and provide reproducible and standardized training opportunities [10, 13, 14, 15]. For instance, a prospective study demonstrated that the Welling scale, applied to 3D‐printed temporal bone models, reliably differentiated between novice residents and experienced otolaryngology residents [13].

Moreover, the lower IQR observed among senior residents suggests reduced variability and increasing consistency in performance with training. This aligns with the principle that practice and experience refine surgical technique and skill consolidation. Seven individual checklist items were significantly different between junior and senior residents, particularly those involving spatial awareness and anatomical boundaries such as the posterior canal wall limit, antrum entry, and sino‐dural angle. In contrast, drilling defect items did not differ significantly between groups. This may reflect the early emphasis on safety and avoidance of iatrogenic injury during training, with residents learning to prioritize structure preservation from the outset. However, alternative explanations should be considered, including potential ceiling effects if most participants avoided major drilling errors or limited sensitivity of the assessment tool to detect subtle differences in drilling technique between groups. Future studies with larger sample sizes and more granular drilling defect metrics may help clarify whether this finding represents true early competency acquisition or a measurement limitation.

The “overall quality” item demonstrated an extremely strong correlation with total performance score (*r* = 0.96), indicating that expert graders' global impressions were closely aligned with objective checklist results. This suggests that overall quality may serve as a meaningful proxy metric in time‐constrained assessment settings, although continued use of itemized checklists remains essential for detailed feedback and skills analysis.

The contrasting groups method yielded a preliminary proficiency score of 45/70, which may serve as a starting point for future validation studies. However, given the limited sample size and absence of accuracy analyses, further multicenter studies with larger cohorts are needed before this threshold can be reliably applied for formal competency assessments. While the small sample size limited the statistical significance of score improvements across repeated attempts, the observed trend of increasing performance on subsequent attempts supports the potential of repeated simulation for skill refinement and learning retention.

In our residency program, performance scores and 3D‐printed temporal bone models are reviewed with residents during their mid‐year and end‐of‐year evaluations with the program director. This allows for structured formative feedback, goal‐setting, and identification of specific skill deficits. Integration of these assessments into ongoing competency‐based evaluations aligns with the broader goals of graduate medical education reform and ensures that progression is based on demonstrated ability rather than case volume alone. Furthermore, the 3D‐printed temporal bone models used in this study cost approximately $10 (USD) per unit in raw materials [10], compared to $400–$700 for cadaveric specimens [16], representing a cost reduction of over 97%.

This study has some limitations that need to be addressed. The sample size, while sufficient for initial validation, may limit generalizability, and additional multicenter data would enhance external validity before widescale adoption as a high‐stakes summative examination. Additionally, residents were not blinded to their scores, as performance feedback is a necessary component of benchmarking skills assessment. Knowledge of prior performance may have influenced motivation and effort on subsequent attempts. Moreover, while the tool showed strong construct validity, further validation studies are needed to confirm predictive validity, such as correlating simulation scores with intraoperative performance. Comparative studies examining skill acquisition and retention between 3D‐printed and cadaveric temporal bone training, as well as their respective transferability to the operating room, would further clarify the role of each modality in surgical education. The non‐significant trend toward improved scores over repeated attempts also suggests a need for longitudinal studies evaluating the impact of deliberate practice between sessions versus testing exposure on long‐term skill improvement. Future work should focus on evaluating transferability of skills from simulation to the operating room.

## 5 Conclusion

The combination of 3D‐printed temporal bones and final product analysis is a reliable method for assessing mastoidectomy surgical skills. It is a low‐cost tool that requires minimal instruction time for scoring and yet produces reliable results. It may be adapted for use in formative and summative evaluations of technical proficiency in otolaryngology training programs.

## Funding

The authors have nothing to report.

## Conflicts of Interest

The authors declare no conflicts of interest.

## Supporting information


**Table S1:** Pearson correlation analysis between checklist items and total mastoidectomy performance score.

## Data Availability

The data that support the findings of this study are available from the corresponding author upon reasonable request.
