# Supplementary material for: Objective Structured Assessment of Technical Skills in Mastoidectomy Using 3D‐Printed Temporal Bones
Source: Laryngoscope. 2026 May 8;136(9):3764–70. doi: 10.1002/lary.70583 (PMC13460529; doi:10.1002/lary.70583)
Supplement: Supplementary file 1 [file LARY-136-3764-s001.docx]

**Supplemental Table S1:** Pearson correlation analysis between checklist items and total mastoidectomy performance score.

| **Checklist Item** | **Correlation** | **Pearson Correlation** |
| --- | --- | --- |
| Posterior canal wall drilling defect | 0.60 | Strong correlation |
| Tegmen drilling defect | 0.71 | Very strong correlation |
| Sigmoid sinus drilling defect | 0.69 | Very strong correlation |
| Semicircular canal drilling defect | 0.83 | Very strong correlation |
| Ossicular chain drilling defect | 0.81 | Very strong correlation |
| Posterior canal wall limit identification | 0.83 | Very strong correlation |
| Tegmen limit identification | 0.82 | Very strong correlation |
| Sigmoid sinus limit identification | 0.82 | Very strong correlation |
| Sino-dural angle identification | 0.72 | Very strong correlation |
| Antrum entered | 0.84 | Very strong correlation |
| Gradual sloping of mastoid cavity edges | 0.75 | Very strong correlation |
| Straightness of mastoid cavity edges | 0.80 | Very strong correlation |
| Appropriate depth of mastoid cavity | 0.88 | Very strong correlation |
| Overall quality | 0.96 | Extremely strong |

Pearson Correlation (r):

0.00–0.30: Weak correlation; 0.30–0.50: Moderate correlation; 0.50–0.70: Strong correlation; 0.70–0.90: Very strong correlation; >0.90: Extremely strong correlation
